# Acrobatics for Antibiotics: Exploring circus-based engagement on community practices surrounding antimicrobial resistance in Cambodia

**DOI:** 10.12688/wellcomeopenres.25343.1

**Published:** 2026-02-11

**Authors:** Rashaad Wijntuin, Moul Vanna, Thyl Miliya, Phallina Thong, Phal Chanpheakdey, Sambou Bran, Abhijit Mishra, Aaryan Dahal, Florine van Driessen, Marco Liverani, Tibor Almasy, Hem Vattanak, Neil Murphy, Elke Wynberg, Rusheng Chew, Mom Ean, Osman Khawaja, Elizabeth M. Batty, Chan Davoeung, Voeurng Bunreth, Kros Sarath, Phaik Yeong Cheah, Sidonn Krang, Dysoley Lek, Paul Turner, Ruth Peters, Bipin Adhikari, Thomas J Peto

**Affiliations:** 1 Vrije Universiteit Amsterdam, Amsterdam, North Holland, The Netherlands; 2 Action for Health Development, Battambang, Cambodia; 3 Cambodia Oxford Medical Research Unit, Siem Reap, Cambodia; 4 Phare Ponleu Selpak, Battambang, Cambodia; 5 Mahidol Oxford Tropical Medicine Research Unit, Bangkok, Bangkok, Thailand; 6 University of Amsterdam, Amsterdam, North Holland, The Netherlands; 7 London School of Hygiene & Tropical Medicine, London, England, UK; 8 N/A Productions, Hong Kong, China; 9 The Fringe, Battambang, Cambodia; 10 Amsterdam Institute for Global Health and Development, Amsterdam, North Holland, The Netherlands; 11 University of Oxford Nuffield Department of Medicine, Oxford, England, UK; 12 Canberra Health Services, Canberra, Australia; 13 Provincial Health Department, Battambang, Cambodia; 14 Provincial Health Department, Siem Reap, Cambodia; 15 Cambodian Ministry of Health, Phnom Penh, Cambodia; 16 National Center for Parasitology, Entomology and Malaria Control, Phnom Penh, Cambodia; 17 University of Health Sciences, Phnom Penh, Cambodia

**Keywords:** Antimicrobial resistance, Antibiotic use, Community engagement, Arts-based engagement, Health education, Qualitative research, Public health, Cambodia, Southeast Asia

## Abstract

**Background:**

Antimicrobial resistance (AMR) is a growing public health threat, particularly in low- and middle-income countries (LMICs) where antibiotic misuse is common and awareness is limited. In Cambodia, misconceptions about antibiotics, such as their use for viral infections and common ailments, are widespread. Culturally relevant strategies are needed to communicate complex health messages to diverse audiences. This study aims to explore the impact of a co-designed, circus-based engagement project on community understanding and perceptions of AMR and antibiotics.

**Methods:**

A qualitative case study design was employed. From May 20 to June 25, 2025, data were collected through 15 Semi-Structured Interviews (SSI) and three Focus Group Discussions (FGD) following performances in Battambang and Siem Reap provinces. Participants represented varied backgrounds and ages (16-60 years). The SSIs and FGDs, conducted in Khmer and translated into English, were complemented by field observations from live performances. All transcripts underwent thematic analysis, and findings are presented based on the research question.

**Results:**

Four key themes were identified: (1) Baseline knowledge and misconceptions ‒ antibiotics were often seen as general-purpose medicines, (2) Community medicine use ‒ treatment-seeking relied heavily on informal sellers, peer recommendations, and often entailed incomplete courses, (3) Audience interpretation of the performances ‒ humour and familiar scenarios supported message recall, though some confusion remained, (4) Key takeaways and intended change ‒ many participants reported plans to seek medical advice from appropriate health services before taking antibiotics and to share correct information with others.

**Conclusions:**

Circus-based engagement was received as a creative and culturally resonant form of health communication that could translate complex biomedical concepts into accessible, and memorable narratives. In Southeast Asia and other LMIC settings with high AMR burdens, such co-designed approaches to knowledge translation could complement conventional education strategies and bridge the gap between scientific knowledge and everyday health practices.

## Plain language summary

This study was carried out by researchers from the Mahidol Oxford Tropical Medicine Research Unit (MORU), the Cambodia Oxford Medical Research Unit (COMRU), Phare Ponleu Selpak, Action for Health Development, with support from the Cambodian Ministry of Health.

The research took place from May 20 to June 25, 2025 in Battambang and Siem Reap provinces, Cambodia. AMR-themed circus-based performances were conducted to see whether circus-based performances could help communities better understand antibiotic use and AMR as it is a major problem in Cambodia, where antibiotics are often misused and awareness is low.

We used a qualitative case study design through interviewing 15 audience members and three focus group discussions after live circus shows. Participants were mostly students, but also included staff, teachers, performers, and a health professional. Performances were also observed by the team members to capture audience reactions.

The study found that circus performances helped audiences reflect on local treatment seeking practices. Humour, familiar scenarios, and metaphors supported recall, and many participants reported intentions to change treatment seeking behaviour. However, some confusion remained about when antibiotics should be used.

These findings suggest that circus-based engagement could complement conventional health education and help people build the skills to make informed health decisions. Creative approaches such as drama-circus can enable communities to seek care more appropriately and use medicines responsibly, helping to reduce AMR in Cambodia and beyond.

## Introduction

AMR is a growing global health threat and is projected to cause millions of deaths annually by 2050 without urgent action, with the greatest burden falling on low- and middle-income countries (LMICs) ^1, 2^. In Southeast Asia (SEA), rising resistance is linked to widespread antibiotic misuse, weak regulation, weak health systems, and gaps in surveillance, leading to increasing treatment failures in both community and hospital settings ^3^.

In Cambodia, misconceptions about antibiotics are widespread ^4– 6^. Antibiotics are frequently used for viral infections such as common colds or influenza, often obtained from pharmacies or informal sellers without prescription ^7– 9^. A 2019 estimate suggested that more than 3,000 deaths in Cambodia were associated with drug-resistant infections, although the actual number is likely to be much higher, as accurately attributing mortalities to AMR remains a major challenge ^2^. These patterns reflect both systemic challenges and limited public understanding of appropriate antibiotic use ^4– 6^.

Conventional health education campaigns, such as posters, lectures, or clinic-based counselling, struggle to reach diverse Cambodian populations due to (health) literacy barriers, linguistic diversity including loss of meaning in technical translation, and limited access in rural areas ^10^. To address these gaps, creative and culturally resonant approaches are increasingly being explored in the region ^11– 13^. Arts-based methods, including theatre, music, and storytelling, have shown promise in promoting awareness and dialogue on health issues such as HIV and malaria in Cambodia and neighbouring countries ^11– 13^. However, their use to communicate abstract and less visible topics such as AMR has received little attention, particularly in LMIC contexts.

This study aims to explore the potential of youth-led circus arts, a vibrant and expressive form of performance in Cambodia, that can communicate messages about AMR and antibiotic use, while also examining audience understanding, existing medicine-use practices, and responses to the performances. To address this aim, the study investigated the following key areas:

1. How do community members interpret the messages about AMR and antibiotic use conveyed through the circus performances?
2. What prior knowledge or misconceptions about AMR and antibiotic use do audience members have?
3. What key messages related to AMR and antibiotic use did audience members take away from the performances?
4. To what extent do audience members report any intention to change their antibiotic-related behaviours as a result of attending the performance?

To our knowledge, this is the first study exploring the use of circus-based engagement on AMR in SEA. By examining how audiences engaged with this less studied but culturally grounded approach, we aim to contribute evidence on innovative strategies for making abstract biomedical concepts more accessible and sustainable in communities where more traditional education methods may fall short ^14, 15^

## Methods

This qualitative case study examined how audiences interpreted and responded to circus performances about AMR and antibiotic use in Cambodia ^16, 17^. Data were collected through SSIs, FGDs, and observational field notes and the study adopted an interpretative-constructivist approach to explore participants’ understanding and lived experiences surrounding medicine use within their social and cultural contexts ^18– 20^. The study followed a COREQ (Consolidated criteria for Reporting Qualitative Research) guideline **(ED1.)** ^21^.

### Intervention: The AMR circus project

This study is part of a larger collaborative public engagement initiative developed in partnership with Phare Ponleu Selpak, a non-profit arts organization based in Battambang and Siem Reap, known for its commitment to social advocacy and creative education ^22^. Each year, Phare provides arts training to over 1,000 students and reaches more than 800 households through its community outreach efforts ^22^.

**Figure 1.**
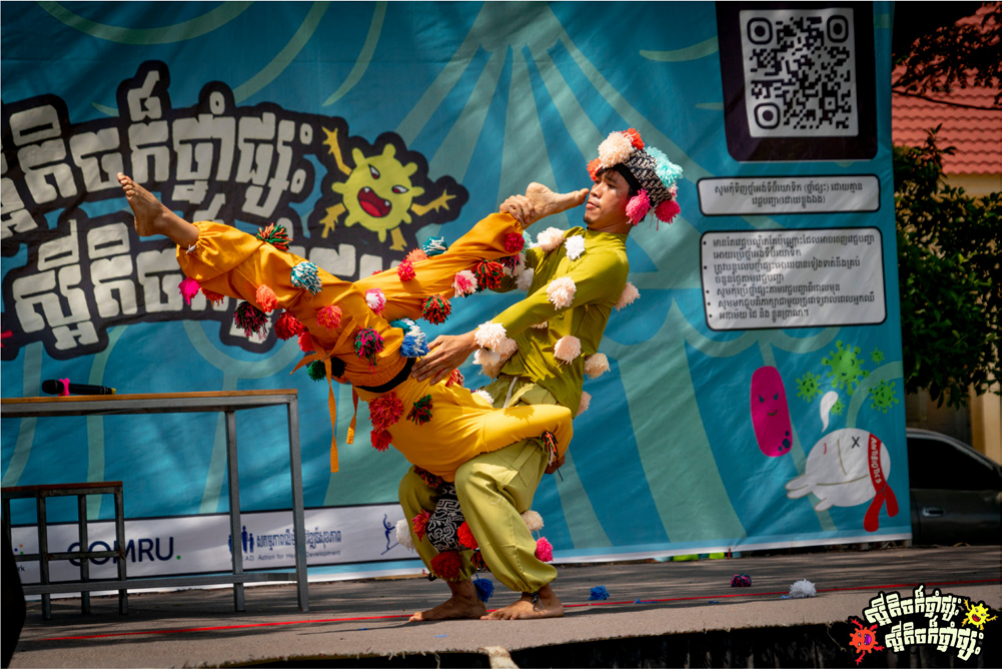
Illustrative show highlight I (Thma Koul performance). Photograph showing the symbolic battle between an antibiotic and resistant bacteria inside the patient’s body during the Thma Koul performance on 20 May 2025. The act used acrobatics and humour to depict how resistance develops when antibiotics are misused. Photo by Nicky Almasy.

**Figure 2.**
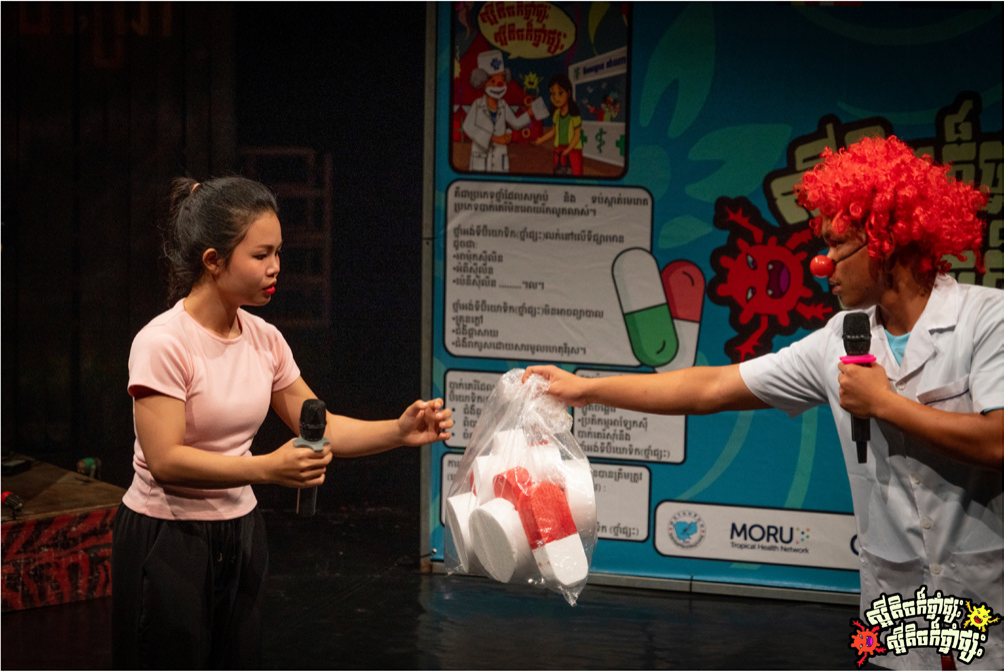
Illustrative show highlight II (Siem Reap performance). A clown pharmacist hands a comically large bag of mixed antibiotics to a patient with a mild cold, illustrating informal over-the-counter sales and community medicine-sharing practices during the Siem Reap performance on 22 May 2025. Photo by Nicky Almasy.

The AMR Circus project was designed by combining acrobatics with narrative storytelling to raise awareness about AMR in Cambodia as a culturally relevant and accessible circus-based education ( Figure 1– Figure 2). The development of the project involved extensive co-creation with a range of local stakeholders, including the National Centre for Parasitology, Entomology and Malaria Control (CNM), the Provincial Health Departments of Battambang and Siem Reap, Phare Ponleu Selpak, Action for Health Development (AHEAD) Cambodia, the Youth Advisory Group on Health and Research Engagement (YAGHRE) ^23^ and researchers from MORU & COMRU.

A core component of the co-design process focused on script development for live circus performances. Local partners provided cultural and experiential insights into community beliefs and practices related to antibiotic use and AMR, allowing the narrative to directly reflect common misconceptions and behavioral patterns in Cambodian communities **(ED2.)**. In addition to the script, a range of other campaign materials were co-designed, including logos, banners, leaflets, posters, questionnaires, t-shirts, and tote bags. Media content was also developed, such as short informative AMR videos featuring the same characters and messages as the circus shows, a recap video series, and a short documentary. These videos, shared through social media, have collectively reached over half a million views, extending the reach of the campaign beyond the physical performances and into digital spaces, particularly among young audiences ^24– 26^.

Between May 19 and May 23, 2025, four performances were held in Battambang and Siem Reap provinces, targeting, but not limited to, youth audiences, who are typically more receptive to creative and interactive education methods ^27^. All performances lasted around an hour and the number of shows was decided based on the logistical and funding feasibility and the audiences were informed through community announcements by local partners and social media posts. Youth audiences are also recognized as an important conduit to their parents and community members, facilitating the dissemination of the messages they have learned. In addition, creative arts students from Phare contributed to the campaign by producing a series of colourful watercolour illustrations that visually translated key AMR messages, further reinforcing understanding and engagement among the attendants.

The AMR Circus Project also gained attention through online local media platforms, amplifying public conversation on AMR and sustaining the relevance of the health messages ^28, 29^. This creative, community-engaged approach demonstrates the potential of performance arts as a powerful medium for wider public health engagement, particularly in contexts where literacy or access to formal health education may be limited.

### Setting

**Figure 3.**
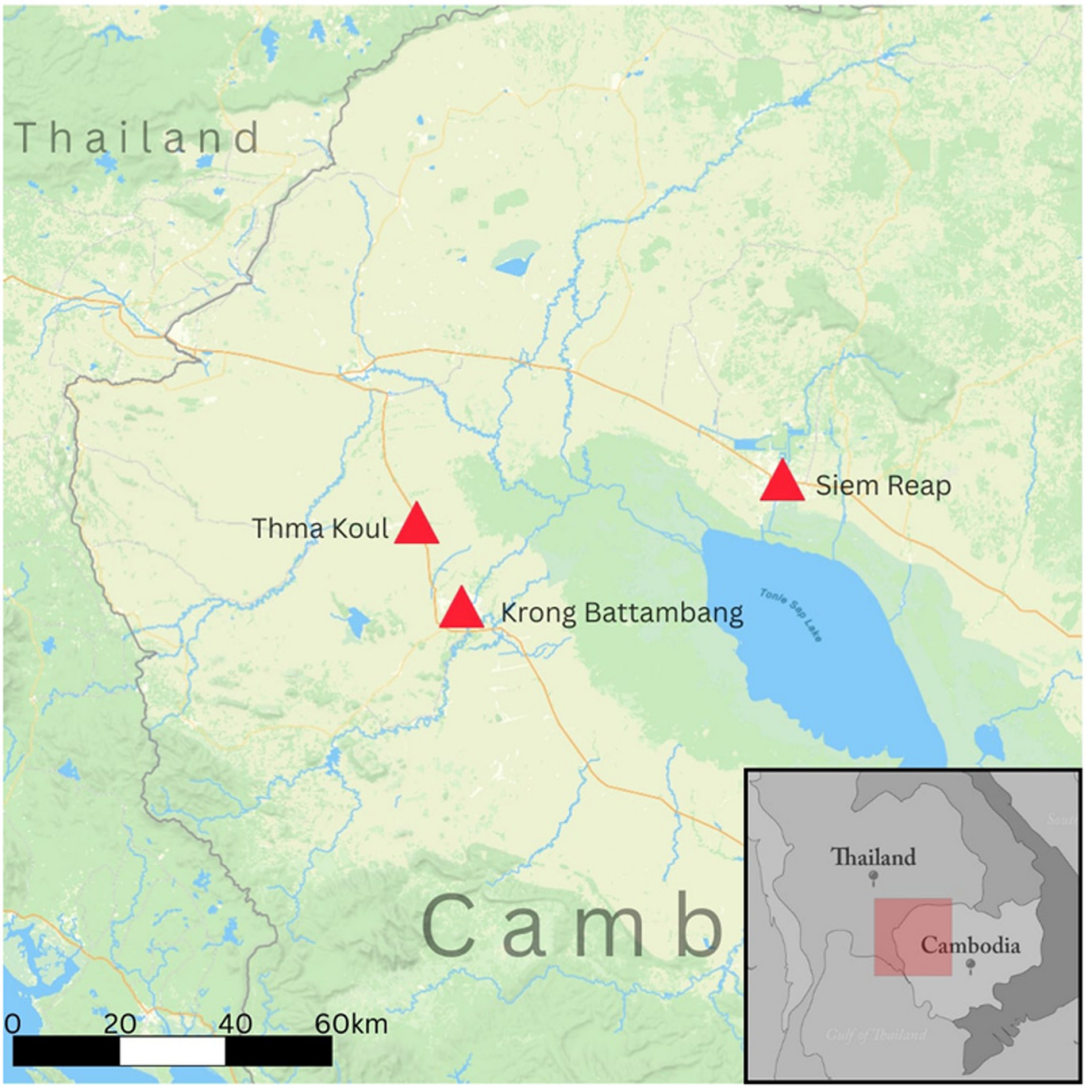
Map of study sites in north-western Cambodia. Map showing the three performance and data-collection locations: Phare Ponleu Selpak (Battambang city), Roung Chrey High School (Thma Koul district), Phare, the Cambodian Circus, and Angkor University (Siem Reap).

Data collection took place across three locations in northwestern Cambodia ( Figure 3) where performances were held. In Battambang, the first show was conducted on the campus of Phare Ponleu Selpak. Although this is an urban venue, audiences consisted mostly of individuals from surrounding rural villages ^30^. The second performance and data collection site was Roung Chrey High School in Thma Koul district, a more rural area within Battambang province ^30^. Two additional performances were conducted in Krong Siem Reap at Phare, the Cambodian Circus, followed by interviews held at Angkor University. These locations were selected to capture perspectives from both urban and rural communities across two provinces ^30^.

### Participants

A total of 15 members of the audience participated in SSIs and 15 in FGDs. After each performance, local research team members approached interested audience members to invite them to take part in the study. Eligible participants were those aged 16 or older who had attended a live performance, with efforts made to include both youth and other community members. All participants were briefed on the study’s purpose, procedures, and confidentiality, and informed that their anonymized perspectives would contribute to the research findings. The number of participants for this study was guided by the principle of data saturation, meaning data collection continued until no new themes or insights emerged from subsequent interviews ^31^.

### Consent

Written informed consent was obtained from all individuals appearing in photographs for the use and distribution of these images in the manuscript and associated online outlets. In addition, written informed consent for participation in the study and for the use of all collected materials in academic publications was obtained from all study participants prior to the interviews and FGDs.

### Data collection

The SSIs and FGDs were conducted in Khmer by a bilingual (Khmer–English) member of the research team (PC) with oversight and support from the lead researcher (RW) from May 20 to June 25, 2025. The interviews lasted between 24 and 65 minutes while the FGDs lasted between 91 and 94 minutes. Interviews followed a flexible interview guide informed by key domains of knowledge, attitudes, and behaviour related to AMR and antibiotic use **(ED3.)**. Participants were asked to reflect on their prior awareness, interpretation of the performance, emotional reactions, and any reported or intended behaviour change. An FGD guide was also developed to explore collective perspectives and group dynamics among audience members. The guide followed the same core domains as the interview guide but encouraged participants to build on each other’s responses, compare interpretations, and discuss shared experiences of the performance **(ED4.)**. Discussions began with informal conversation and basic demographic questions, followed by open-ended prompts. To supplement verbal data, observational field notes were recorded during three circus performances, two in Battambang and one in Thma Koul, by (RW). A second team member (BA) recorded two field notes in Battambang and two in Siem Reap. Field notes captured the structure and content of the shows, audience engagement, their reactions, general setting of the shows, and delivery of AMR messages. These observations provided additional context to support interpretation of how the performances were received ^19^.

### Data analysis

All interviews and FGDs were audio recorded, transcribed verbatim in Khmer, and translated into English by a professional translation service. Transcripts were cross-checked for accuracy prior to analysis. Thematic analysis was conducted using the six-phase approach described by Braun and Clarke ^32^. Data were first read for familiarization, followed by the generation of initial codes. Codes were then organized into potential themes, which were reviewed, defined, and refined through iterative analysis and discussion among team members (RW, BA and PC). Both deductive and inductive approaches were applied for coding. Deductive coding derived from the interview guide focused on areas relevant to the study’s objectives, including prior knowledge of AMR, interpretation of performance messages, and behavioural intentions. Inductive coding allowed for emerging insights to develop, including emotional responses to the performance or social factors shaping understanding. Coding was conducted line by line using ATLAS.ti 25 software ^33^ and themes were developed through a framework of parent and child codes **(ED5.)**. Observational notes were integrated into the thematic framework to enrich the analysis and support reflexivity in interpreting audience responses.

### Reflexivity

RW, a researcher from the Netherlands, led the study in collaboration with BA, a Nepalese social scientist with extensive experience conducting community-based qualitative research in Southeast Asia. Interviews were conducted in Khmer by PC, a local researcher and translator familiar with the community context. Regular discussions between RW, BA, and PC throughout the study helped to reflect positionality, cultural interpretation, and the influence of researcher backgrounds on data collection and analysis.

### Public involvement

Local stakeholders were actively involved in the development of the engagement activities. Staff from the local partner organisations, circus staff and members of a local youth group contributed to shaping the storyline of the circus-drama performances and provided input into the design of printed materials during the project’s development phase. Their contextual knowledge and lived experience informed how AMR-related messages were integrated into the performances and supporting materials. Participants and community members were not involved in the methodological design of the research study. However, feedback collected through interviews and discussions informed adaptations to subsequent performances. All data collection procedures, research questions, and analytic approaches were designed by the research team without direct involvement of patients or the public.

### Ethics statement

This study received ethical approval from the University of Oxford Tropical Medicine Research Ethics Committee (OxTREC), Oxford, UK, and the Cambodian National Ethics Committee for Health Research (reference #061 NEHCR) on February 06, 2025. All procedures involving human participants were conducted in accordance with the Declaration of Helsinki.

## Results

The sociodemographics of the participants are presented in Table 1

**Table 1.** Sociodemographics and data-collection details of study participants (n = 30). Summary of participant age, sex, occupation, residence, show location, interval since performance, and data-collection method (SSI or FGD).

Participant	Age	Sex	Occupation	Residence	Show	Days from show	Data collection method
1.	16–20	F	Student	Urban	Krong Battambang	7	SSI
2.	20–30	F	Student	Urban	Siem Reap	7	SSI
3.	20–30	F	Student	Rural	Krong Battambang	8	SSI
4.	40–50	F	Teacher	Urban	Krong Battambang	8	SSI
5.	20–30	F	Student	Rural	Siem Reap	7	SSI
6.	16–20	F	Student	Rural	Thma Koul	8	SSI
7.	16–20	F	Student	Rural	Krong Battambang	7	SSI
8.	20–30	F	Staff	Urban	Krong Battambang	9	SSII
9.	16–20	M	Student	Rural	Thma Koul	8	SSI
10.	50–60	F	Health professional	Rural	Thma Koul	8	SSI
11.	20–30	F	Staff	Rural	Krong Battambang	1	SSI
12.	20–30	M	Student	Rural	Krong Battambang	1	SSI
13.	16–20	M	Student	Rural	Thma Koul	8	SSI
14.	20–30	F	Student	Rural	Krong Battambang	8	SSI
15.	20–30	F	Student	Rural	Krong Battambang	10	SSI
16.	20–30	F	Performer	Rural	Krong Battambang	28	FGD
17.	16–20	M	Student	Rural	Thma Koul	33	FGD
18.	20–30	M	Performer	Urban	Krong Battambang	28	FGD
19.	16–20	F	Student	Rural	Thma Koul	33	FGD
20.	16–20	F	Student	Rural	Thma Koul	33	FGD
21.	16–20	F	Student	Rural	Thma Koul	33	FGD
22.	20–30	F	Student	Urban	Krong Battambang	28	FGD
23.	16–20	F	Student	Rural	Thma Koul	33	FGD
24.	16–20	F	Student	Rural	Thma Koul	33	FGD
25.	20–30	M	Performer	Urban	Krong Battambang	28	FGD
26.	20–30	F	Performer	Urban	Krong Battambang	28	FGD
27.	16–20	F	Student	Rural	Thma Koul	33	FGD
28.	20–30	F	Student	Rural	Krong Battambang	28	FGD
29.	20–30	M	Student	Urban	Krong Battambang	28	FGD
30.	16–20	F	Student	Rural	Krong Battambang	28	FGD

**Figure 4.**
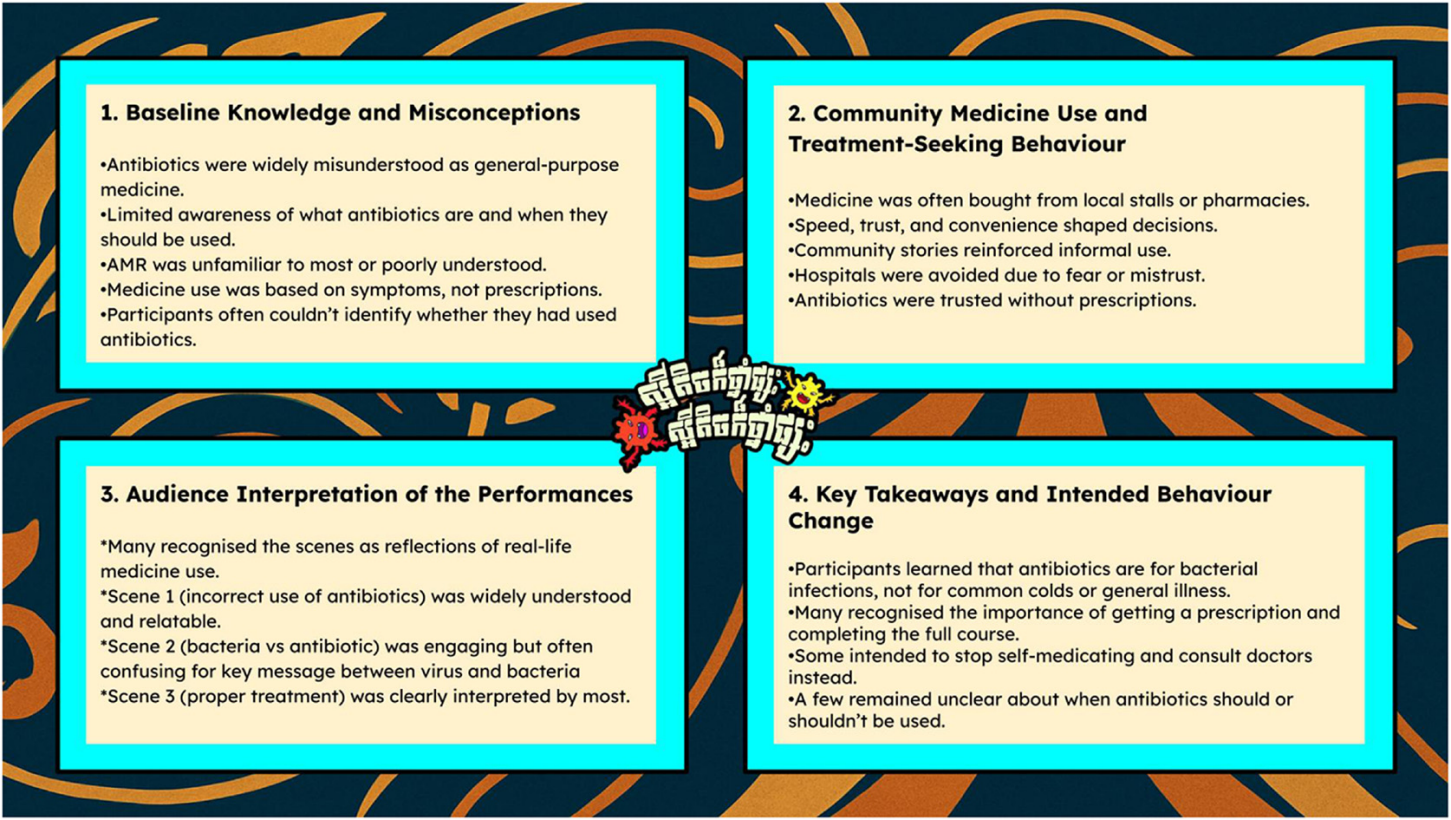
Thematic domains identified from qualitative analysis. Illustration summarising the four main thematic domains derived from interviews and focus groups: baseline knowledge, community medicine use, audience interpretation of performances, and intended behaviour change.

Thematic analysis of 15 SSIs and three FGDs yielded four key domains that address the research questions. These key domains, presented in Figure 4, were supported by illustrative participant quotations.

### 1. Baseline knowledge and misconceptions

***Participants’ previous knowledge of antibiotic use.*** Participants mentioned limited awareness and several misconceptions regarding antibiotics before the performances. Most associated antibiotics with general-purpose medicine, often perceived as effective for treating a wide range of common ailments. The term “antibiotic” was familiar to some but poorly understood in terms of its specific functions or limitations.

Many participants believed antibiotics could be used to treat nearly any illness even for minor symptoms.

*“Well, before the show, I thought antibiotics could be used for anything. Even if you have a small headache, it can be used. Because at first, I thought the more you use it, the better it will become.”* (P2, SSI, 20–30 years, Siem Reap).

***Participants’ previous knowledge about AMR.*** When asked about AMR, most participants stated that they had not heard of the term before and among those who had, knowledge was often vague or incomplete. A number of participants described situations in which a medicine worked less effectively after repeated use, which they associated with the idea of resistance.

*“Yes, I used to hear about it [AMR]. I heard, like, it was cured when we took it for the first time, but when we took it for the second or third time, it became addictive to our body, and there was no more effect.”* (P1, SSI, 16–20 years, Krong Battambang).

***Participants’personal experiences using medicine.*** Participants reflected on their past experiences with using medicine to treat common illnesses such as sore throats, febrile illnesses, and toothaches. Several explained that they were often unable to recall whether they had used antibiotics specifically, as medicines were commonly received in unlabelled combinations prepared by pharmacists or recommended by family members. These mixtures were typically provided based on common symptoms rather than prescriptions, and their exact compositions were not always known. Despite this uncertainty, some participants mentioned specific antibiotics they had used in the past, often for non-specific conditions.

*“Usually, if it’s mild, I just try home remedies or rest. But if it doesn’t get better after a few days, I go to the pharmacy to get medicine. I just tell them my symptoms, and they give me some medicine, I take it without really knowing what it is.”* (P12, SSI, 20–30 years, Krong Battambang).
*“I have sometimes used it when I got a sore throat; I used antibiotics. I also used antibiotics when I got a toothache or something sharp. I mostly use Clamoxyl or Amox [Amoxicilline]. In our country, we normally use them.”* (P4, SSI, 40–50 years, Krong Battambang).

### 2. Community medicine use and treatment-seeking behaviour

***Local medicine sources.*** Participants described medicine use as part of the fabric of their treatment seeking behavior, shaped by severity, trust in local providers, and the influence of shared (communal) experiences. Seeking treatment from local pharmacies, stalls, or informal providers was often the preferred approach, especially for common symptoms such as stomach pain. These practices were seen as convenient and effective, offering quick recovery without the need for formal consultation and the perceived hassle associated with public healthcare services.

*“They want to recover very quick. So, they don’t want to do the checkup. They don’t want to wait for the service at their hospital. They just go and give [the pharmacist] money and get the medicine. And the medicine even gives them the very fast result. And they trust the pharmacist.”* (P8, SSI, 20–30 years, Krong Battambang).

***Community influence.*** The treatment seeking practices were often reinforced through community storytelling and intergenerational memory. One participant reflected on how the reputation of antibiotics as a “miracle medicine” was passed down from the wartime experiences of older generations:

*“.....my mom told me [she] was in the war, and mostly people got sick, like hurting, from the shootings and bombs. When they went to the health center, they used antibiotics to keep them well and better. After that, they still think antibiotics are like the angel of medicine … the best one that can recover all disease.”* (P15, SSI, 20–30 years, Krong Battambang).

The influence of peers and word-of-mouth was another key factor. Medicines that appeared to work for one person were often recommended to others. In contrast, hospitals were often viewed with scepticism or fear, and avoidance of public healthcare was described as a common community practice.

### 3. Audience interpretation of the performance

**Figure 5.1.**
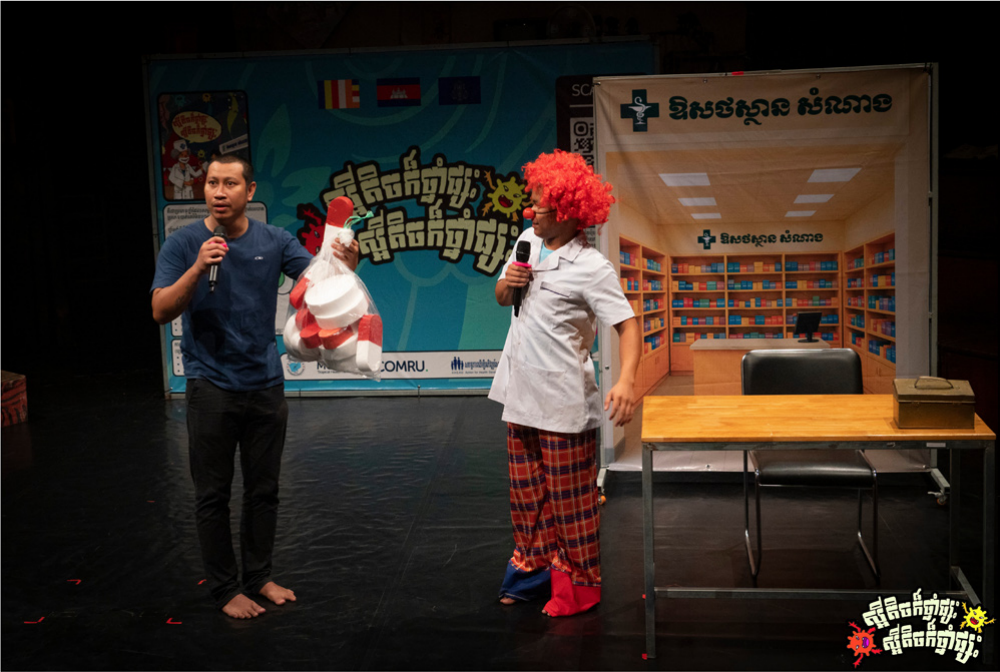
Scene one: Welcome to Samnang’s pharmacy (part I). Depicts the chaotic opening of Samnang’s Pharmacy, where antibiotics are sold indiscriminately and in large combined bags for little money, highlighting the prevalence of informal, non-prescription sales in Cambodia. Photo by Nicky Almasy.

**Figure 5.2.**
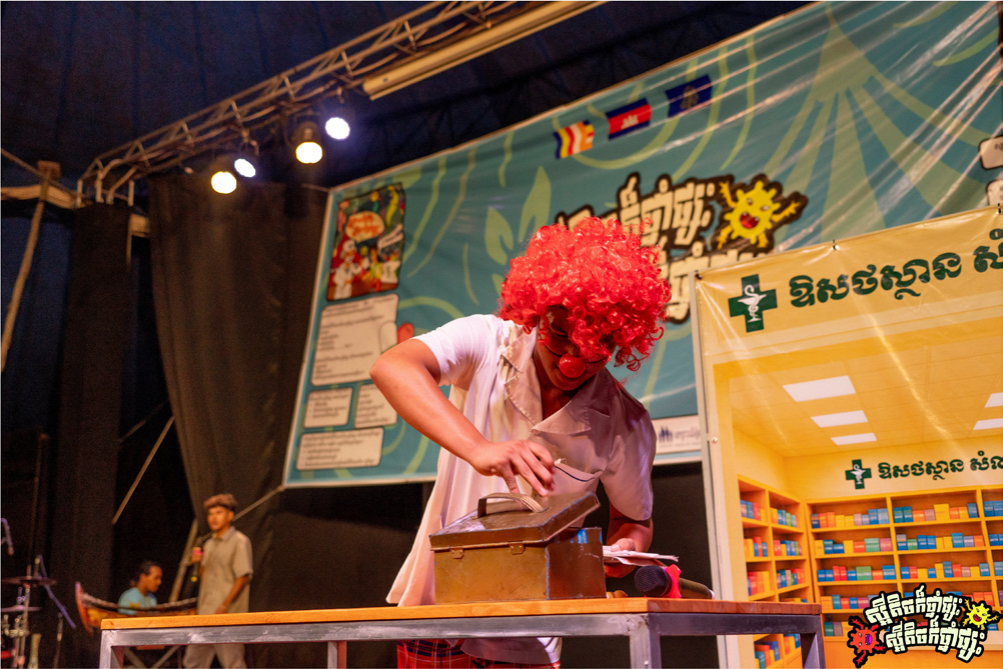
Scene one: Welcome to Samnang’s pharmacy (part II). Shows part of the scene where the clown pharmacist (Samnang) searches his pill box for random drugs to give to clients. Photo by Nicky Almasy.

***Scene one: Welcome to Samnang’s pharmacy.*** Scene one of the circus-drama depicted a clown pharmacist who opened his shop by juggling pills carelessly, without paying attention to what they were ( Figure 5.1– Figure 5.2). Three patients approached him one by one; a girl with a cold, a man with flu, and another with a stomachache. Without asking any questions or checking symptoms, the clown pharmacist handed each of them a large bag of mixed antibiotics. The first patient told the second, and the second told the third, forming a chain of recommendations. The scene was designed to highlight casual medicine selling, Over-The-Counter (OTC) access to antibiotics, and how word-of-mouth can reinforce OTC use of antibiotics.

Several participants recognized the scene as a reflection of what happens when people self-medicate without proper diagnosis or professional guidance. Participants explained that the medicine being given was inappropriate for the range of symptoms the patients presented and for some, the scene stood out as the most memorable part of the performance because it closely mirrored current community practices and personal experiences. The influence of social recommendation and the reputation of medicine sellers also featured in some participants’ interpretations. A participant described how trust in a well-known local seller, reinforced by community rumours, often leads people to bypass formal healthcare entirely:

*“The scene that resonated with me was the first one, where they went to buy medicines without diagnosis. It reminded me of how I always used medicine improperly when I got sick and didn’t go to the hospital. I really regret it, because it made my health worse. I had problems with my kidneys and stomach aches from taking too much medicine, and my health became weak because of that.”* (P24, FGD, 16–20 years, Thma Koul).

**Figure 6.1.**
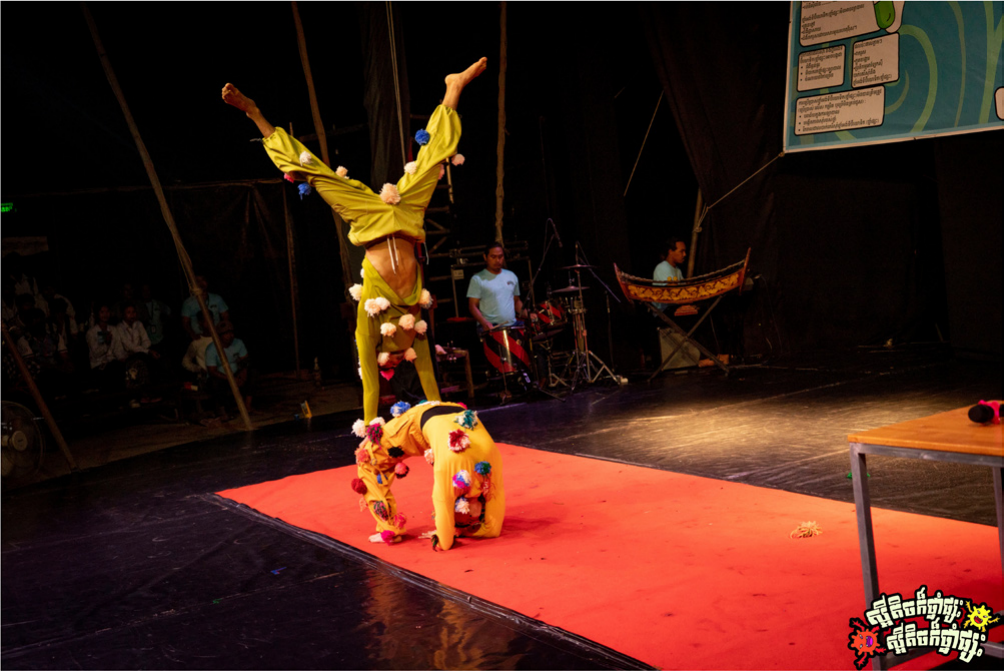
Scene two: The showdown, bacteria vs. antibiotic (part I). Illustrates the confident antibiotic character defeating bacteria in the first round, symbolising the short-term effectiveness of medicine after misuse. Photo by Nicky Almasy.

**Figure 6.2.**
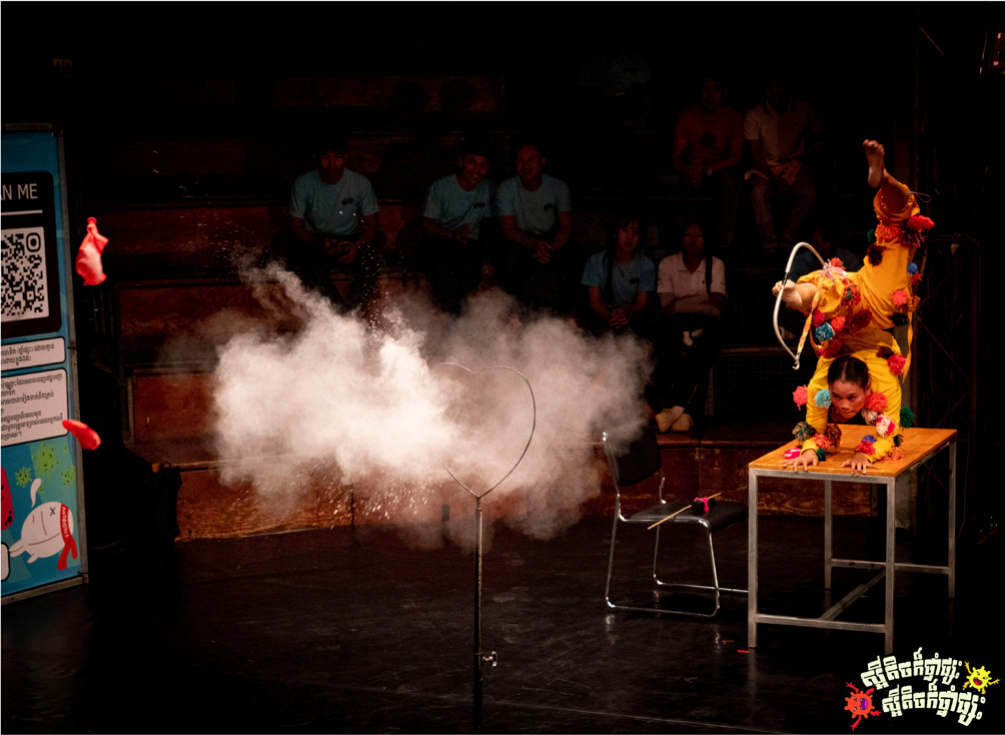
Scene two: The showdown, bacteria vs. antibiotic (part II). Shows the bacteria returning stronger and bursting the antibiotic’s heart-balloon using a bow and arrow with the feet in an acrobatic stance, a symbolic representation for resistance overcoming the strength of the antibiotic. Photo by Nicky Almasy.

***Scene two: The showdown, antibiotic vs. bacteria.*** Scene two of the circus performance portrayed the battle between antibiotics and bacteria inside the patients’ bodies ( Figure 6.1– Figure 6.2). The antibiotic character began with confidence, defeating the bacteria easily and boasting about his strength. However, the bacteria returned stronger and, in a humorous and acrobatic sequence, shot an arrow that burst the antibiotic’s protective balloon. The antibiotic, now weakened, exited the stage dramatically. Observations suggested that it was consistently the most engaging segment of the circus-drama, with the audience responding visibly to its humour and symbolic storytelling to represent the growing threat of antimicrobial resistance, second only to the final circus acrobatics in terms of attention and enthusiasm.

These observations were reflected in the participants’ responses when asked if they could recall the scene and its key message. A few participants clearly interpreted the scene as showing resistance developing due to incorrect use or insufficient dosage. Most of them explained that while antibiotics might help at first, their power weakens over time if misused:

*“Drug resistance is when the bacteria or virus knows the ability of the medicine and it continues to spread in our body and makes our disease even more serious and the bacteria will become stronger…..even if we take antibiotics repeatedly, it won’t have ability to overcome disease anymore, and it will make it difficult to find suitable medicine to fight against the bacteria.”* (P6, SSI, 16–20 years, Thma Koul).

However, confusion around the characters and the message was sometimes observed, particularly in distinguishing bacteria from viruses. Several participants referred to the antagonist as a virus rather than a bacterium.

Although the performance effectively used visuals and humour to convey the idea of weakening medicine, the specific concept of antimicrobial resistance against bacteria was not always clearly distinguished from more general ideas about medicine versus illness.

**Figure 7.1.**
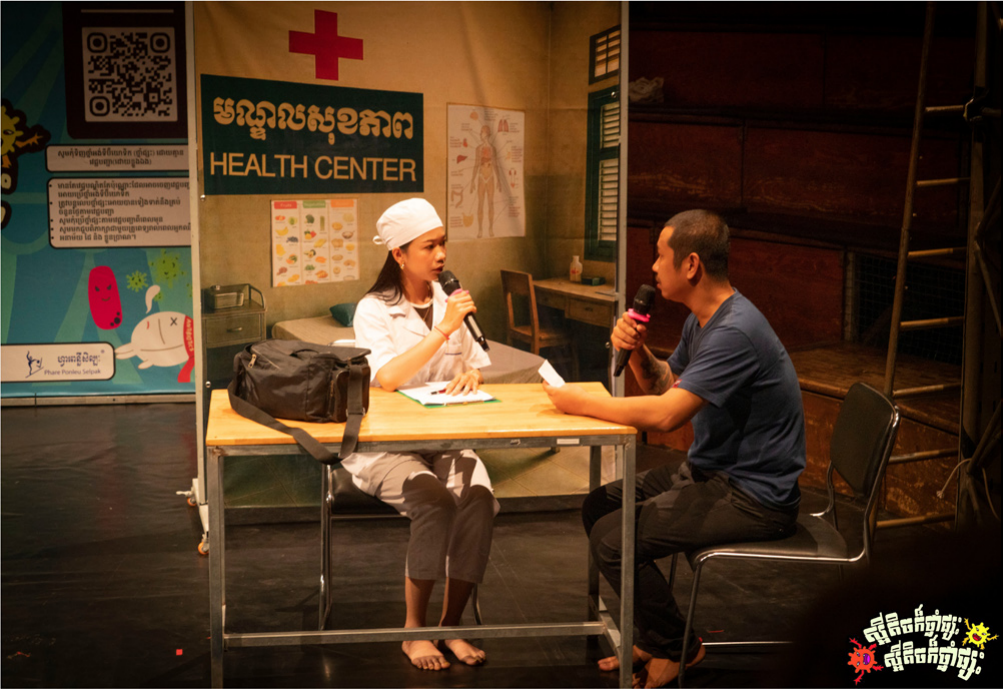
Scene three: The health professionals take the stage (part I). Depicts a doctor and assistant examining returning patients and offering correct prescriptions through formal consultation. Photo by Nicky Almasy.

**Figure 7.2.**
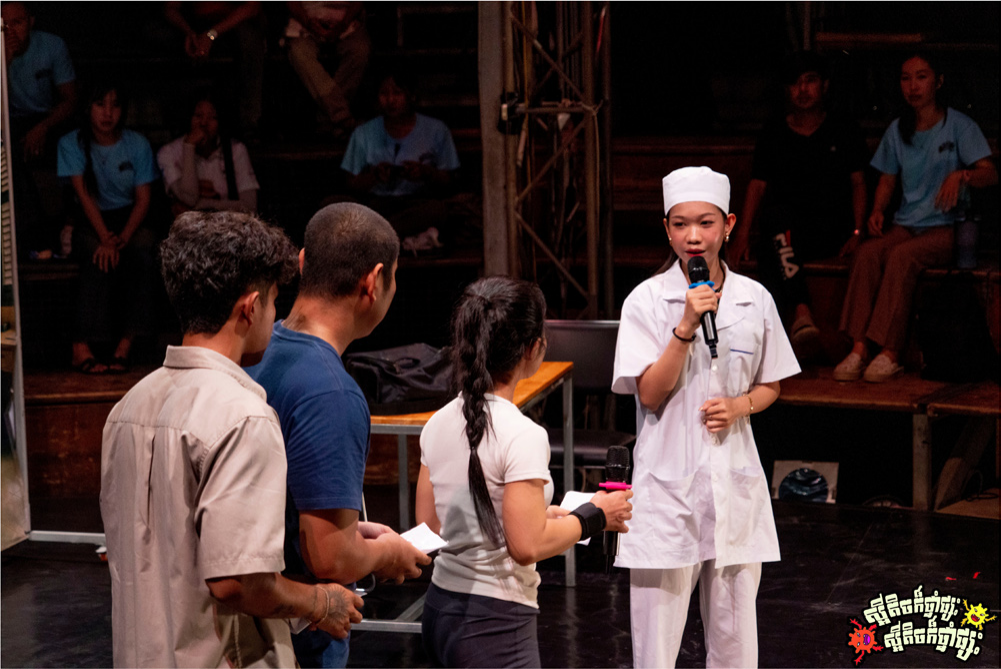
Scene three: The health professionals take the stage (part II). Shows patients receiving education from the health professional regarding AMR, its consequences and responsible medicine use. Photo by Nicky Almasy.

***Scene three: Health professionals take the stage.*** Scene three depicted the moment when the patients, having become more ill after self-medicating with antibiotics, turned to the health centre for care ( Figure 7.1– Figure 7.2). This time, the doctor and her assistant provided them with accurate guidance and appropriate prescriptions, emphasizing that antibiotics should not generally be used for illnesses such as colds, sore throats, and stomachaches in the first instance without medical advice.

Many participants interpreted the scene with a high level of accuracy with regards to the correct practices of using antibiotics, describing both the storyline and the intended key messages, acknowledging the fact that the interviews were conducted within days or weeks of the shows. Several recognized the contrast between the earlier informal medicine use and the advice offered at the health centre.

*“It showed the patients going back to the clinic after the medicine didn’t work. They realized they needed proper treatment, because [their] symptoms are usually caused by viruses, not bacteria. Antibiotics only work against bacteria. If we use them anyway, we also kill good bacteria in our bodies.”* (P12, SSI, 20–30 years, Krong Battambang).

While many participants described the scene in line with the key messages, one participant acknowledged that although the performance offered guidance, they still felt unsure about the specific uses of antibiotics.

*“I’m still not clear which diseases we should use antibiotics for, and which diseases we should not use antibiotics.”* (P3, SSI, 20–30 years, Krong Battambang).

These accounts suggest that while scene three was widely interpreted as a call for proper diagnosis and responsible antibiotic use, some uncertainty remained among participants about specific indications for antibiotics. Nonetheless, the scene’s message appeared to resonate, particularly in contrast with the informal treatment-seeking shown earlier in the performance.

### 4. Key takeaways and intended behaviour change

***Key takeaways.*** Participants were asked what messages they took away from the performance. Many highlighted the importance of using antibiotics only when necessary and with proper medical guidance, noting the risks of self-medication and the need for clear prescriptions. They showed an awareness that misuse or overuse can reduce the effectiveness of antibiotics over time while increasing the treatment costs. Several participants also recalled specific lessons from the performance, such as the difference between bacterial and viral infections, the need for stronger medicines when resistance develops, and the importance of completing prescribed doses. Overall, participants recognized that while antibiotics can be helpful, they can also cause harm if used without professional medical advice, for instance when using OTC antibiotics for illnesses that do not require them.

*“...after watching the show, I think that medicine is a two-sided weapon that has both advantages and disadvantages, but to prevent us from getting the disadvantages we should go to see a doctor who has real expertise at health centers, or at various pharmacies that have doctors with clear prescriptions.”* (P6, SSI, 16–20 years, Thma Koul).

***Remaining uncertainties about antibiotics.*** However, a few participants were still not confident in explaining what antibiotics are used for. In several cases, participants offered vague or approximate definitions or stated that they still did not fully understand the term. One participant acknowledged not knowing what specific medicines antibiotics referred to, and offered a general explanation focused on recovery.

*“For antibiotics, I don’t know what medicine they refer to. I don’t quite understand that, it’s not clear yet. Antibiotics are the medicines that we take to, uh, treat the disease. To make the disease recover quickly.”* (P5, SSI, 20–30 years, Siem Reap).

Participant two attempted to explain that antibiotics are not effective against symptoms caused by viruses but suggested that paracetamol can fight those bacteria.

*“…antibiotics are a type of medicine that cannot get rid of bacteria that are from a virus, a cold, fever, or headache. Only paracetamol can, uh, fight against those bacteria.”* (P2, SSI, 20–30 years, Siem Reap).

These responses suggest that while the performance helped most of the participants engage with new concepts around medicine use, clear and consistent understanding of what antibiotics are, and how they differ from other medicines, remained limited for some individuals.

***Intentions to change medicine use.*** Participants were asked whether they would do anything differently after watching the performance. While many described what they had learned, some explicitly discussed changes in behaviour or intentions to change in the future. They mostly implied that the performance prompted them to seek medical advice from trained professionals rather than relying on medicine sellers. The responses suggest an emerging awareness of the risks of antibiotic misuse and a willingness to change health-related behaviour.

*“After watching the show, it changed my thoughts about disease… meaning that when I’m sick, I must go see a doctor to get checked up before using medicines… I have stopped going to ask the pharmacist, because mostly they only ask the symptoms, they don’t know our actual disease.”* (P3, SSI, 20–30 years, Krong Battambang).

***Advice to the community.*** In addition to describing what they had learned for themselves, some participants also discussed how they had already shared this information with others or planned to do so. These responses often reflected a sense of personal responsibility to help others understand the importance of using antibiotics appropriately and seeking professional medical advice. Advice to family, friends, and neighbours tended to emphasize the dangers of improper use and the benefits of consulting a doctor or visiting a clinic. Participants described encouraging their relatives to seek prescriptions from trained professionals and warning friends about the risks of taking medicines without knowing if they were appropriate:

*“I told my family about the usage of antibiotics and what we should do in order for the usage to be effective and help prevent our health from deteriorating or [be] faced with more diseases. For antibiotics, if we want to use it correctly for our disease, we have to go to a health center or a hospital that is near our home, and we have to ask about the prescription from the doctor.”* (P6, SSI, 16–20 years, Thma Koul).
*“I chatted with friends about using medicines correctly according to our disease… I explain to them about the consequences if they don’t use it correctly. It will make our disease even more serious, and make us waste a lot of money.”* (P9, SSI, 16–20 years, Thma Koul).

## Discussion

This study explored how a co-designed circus performance influenced community understanding and perceptions of antibiotic use and AMR in Cambodia.

Many participants entered the performances with limited understanding of antibiotics and AMR, reflecting common misconceptions seen across Cambodia and other LMICs ^5, 6, 34, 35^. The circus offered an engaging way for audiences to reflect on their existing perceptions of antibiotics. It helped prompt recognition that antibiotics are not a cure for all illnesses, though some confusion persisted about appropriate use. This indicates that while the performance encouraged reflection and partial shifts in understanding, sustained engagement is needed to reinforce accurate knowledge and practice.

Second, the results revealed how deeply antibiotic use is embedded in local community practices. Convenience, trust in local medicine sellers, and advice from family or neighbours shaped treatment-seeking behaviour. A notable detail that emerged was the intergenerational reputation of antibiotics in the community, described as the best medicine which can cure all disease. This was shaped by the experiences of previous generations who, during times of conflict and the collapse of the healthcare system, faced severely limited access to treatment ^36^. These circumstances encouraged habits of self-medication and reliance on OTC antibiotics, which continue to influence treatment-seeking behaviours today ^36^. This reiterates the importance of targeting youth, as this stage offers the greatest potential for lasting behavioural change ^27^. Adolescence is a formative period when habits and decision-making patterns are still developing, making young people more receptive to new ideas and social influence. Early engagement can therefore help correct misconceptions and prevent entrenched behaviours from carrying into future generations ^37^. In Bangladesh, antibiotic practices are often shaped by family traditions and social advice, with younger generations adopting patterns from parents and elders rather than formal health sources ^38^. Similarly, in Vietnam, limited access to trustworthy health information leads many to rely on local networks and informal drug sellers ^34^. In such contexts, intergenerational habits strongly shape antibiotic use, making behaviour change through conventional health education challenging ^34, 38^. The narratives collected here build on this evidence by illustrating how community experiences and shared practices continue to reinforce current treatment-seeking behaviours.

Scene one effectively illustrated antibiotic practices in the Cambodian context. Many participants went beyond describing what they saw, using the scenes as a reflection on their own or their community’s medication habits. This indicates a level of critical engagement with the content, rather than the passive entertainment alone. Research in Cambodia has shown that placing health messages within familiar artistic forms can strengthen community ownership and make them more relatable ^11^. In line with this, our findings suggest that humour and local storytelling potentially allowed for better message recall while also encouraging reported intentions to change behaviour. Evidence from other contexts supports this pattern, for example, educational theatre in the UK has also been shown to improve public understanding of AMR ^27^. A central concern in this study was whether circus arts could effectively communicate a complex and relatively abstract health concern such as AMR in an LMIC setting. The findings seem to show that by drawing on co-creation with local communities, the performances achieved cultural resonance that made the information both accessible and memorable ^39^. Taken together with evidence from other contexts, this project demonstrates that circus arts have potential to become a powerful tool for conveying complex health messages across diverse settings, with particular promise in LMIC contexts.

The humorous acrobatics and metaphors in Scene two made the idea of antibiotics losing strength due to misuse both engaging and easy to recall, though some confusion between bacteria and viruses remained. Similar creative initiatives, such as the WHO’s Don’t You Dare project, have achieved comparable success in engaging youth audiences ^40^. Our findings show that entertaining health communication on AMR also resonates strongly with rural communities ^41^.

Scene three showed the consequences of self-medicating, and participants recalled the messages from this scene with notable clarity, often citing it as the main source of their take-home lessons and intentions. However, whether these reported intentions will translate to behavioural change remains a topic of future study. The effectiveness of drama theatre in conveying health messages has been shown in Cambodia before, for example through village dramas on malaria, as well as in other LMIC settings where improved comprehension and recall was reported ^11, 13, 42^. What distinguished our study was the balance between humorous, acrobatic scenes that captured attention and serious, dramatic moments that reinforced the significance of antibiotic misuse. When learning is entertaining, audiences are more attentive and receptive, allowing key messages to settle in through curiosity rather than obligation ^43^. Storytelling, humour, and performance tap into emotions and social connection, turning abstract ideas into experiences that feel relevant and personal ^41, 43^. These emotional experiences also strengthen memory and recall, as people are more likely to remember what they’ve laughed at, related to, or felt moved by. In this way, entertainment not only captures attention in the moment but helps messages endure long after the event has ended. This lasting recall is what gives arts-based engagement its sustainability in health education, transforming performances from temporary awareness efforts into ongoing sources of reflection and community dialogue ^43, 44^.

### Strengths and limitations

This study has several strengths. To our knowledge, it is the first to evaluate a circus-based AMR engagement project in a Southeast Asian setting. The co-designed nature of the intervention with local artists and researchers, and the triangulation of data from observations, SSI, and FGD provided a rich exploration of community perceptions. Including diverse participants across urban and rural sites offered a broader view of community responses.

However, the small, purposively selected sample may have introduced self-selected bias and limits generalizability ^45^. Furthermore, without baseline data or follow-up, it was not possible to assess knowledge change, message retention, or whether reported intentions led to sustained behaviour. Some confusion also remained about which illnesses require antibiotics, suggesting that while misuse was effectively illustrated, correct indications were less clearly conveyed.

### Future Research and implications

Despite these limitations, circus-based performances show strong potential as culturally resonant tools for AMR engagement in LMICs. However, they are resource-intensive and may be challenging to scale in low-resource settings. Their greatest value may lie in serving as a focal point within broader campaigns that combine live performance with more scalable, lower-cost approaches such as social media, posters, and school-based programmes. This approach aligns with a wider trend of experimenting with creative methods to address AMR. Around the world, researchers and practitioners are increasingly turning to arts-based engagement to make abstract concepts more tangible and memorable such as the Superheroes Against Superbugs project in India which used comics, short animations, and school workshops to raise awareness among adolescents, showing measurable improvements in knowledge and recall ^46^. Together with our findings, this highlights an emerging scientific area that recognizes creativity and cultural resonance as vital for making AMR accessible and memorable. Future studies should build on this momentum by including larger and more diverse populations, baseline and follow-up assessments, and mixed-methods designs to capture both knowledge and behaviour change ^47^. Longitudinal research is especially important to assess message retention and sustained impact ^48^. Comparative studies against conventional health education strategies would provide critical evidence on the unique contribution of creative engagement to AMR communication.

## Conclusion

This study provides promising evidence that circus-based engagement can influence community understanding of antimicrobial resistance and proper antibiotic use in Cambodia. While not without challenges, such as limited scalability and knowledge retention concerns, circus-based health communication holds considerable potential in LMIC settings. Co-designed, locally relevant interventions that combine creativity with clear messaging can bridge the gap between biomedical concepts and everyday understanding, particularly when addressing abstract yet urgent public health threats like AMR.

## Data Availability

Data are available under the terms of the Creative Commons Attribution 4.0 International license (CC-BY 4.0). Because of the nature of the qualitative data in this study, even if the data are anonymized, potential respondents are identifiable based on contextual details. Both MORU and the local ethics committee restrict the sharing of data that can potentially identify the respondents and permit data sharing only on a case-by-case basis. The data is available upon request to the Mahidol Oxford Tropical Medicine Research Unit Data Access Committee ( datasharing@tropmedres.ac). Access may be granted to qualified researchers for ethically approved purposes, following review of the request and the signing of a data access agreement. Only de-identified data will be provided, and access will be subject to the conditions stipulated by MORU and the local ethics committee. ( https://www.tropmedres.ac/units/moru-bangkok/bioethics-engagement/data-sharing/moru-tropical-network-policy-on-sharing-data-and-other-outputs). **(ED1.)** COREQ checklist: https://doi.org/10.6084/m9.figshare.30600671.v1 ^49^. **(ED2.)** Performance description: https://doi.org/10.6084/m9.figshare.30604445.v1 ^50^. **(ED3.)** SSI guide: https://doi.org/10.6084/m9.figshare.30604484.v1 ^51^. **(ED4.)** FGD guide: https://doi.org/10.6084/m9.figshare.30604493.v1 ^52^. **(ED5.)** Coding tree: https://doi.org/10.6084/m9.figshare.30604499.v1 ^53^.
