# Supplementary material for: Acrobatics for Antibiotics: Exploring circus-based engagement on community practices surrounding antimicrobial resistance in Cambodia
Source: Wellcome Open Res. 2026 Feb 11;11:104. [Version 1] doi: 10.12688/wellcomeopenres.25343.1 (PMC13122148; doi:10.12688/wellcomeopenres.25343.1)
Supplement: Peer review history

# Reviewer report: Reviewer response for version 1

**DOI:** 10.21956/wellcomeopenres.27927.r153004

**Published:** 2026-04-28

**Authors:** Vijay Singh Gondil

**Affiliations:** 1 University of Rochester Medical Center, Rochester, New York, USA

The article appears well-designed and underscores the dire need for AMR information dissemination programs. The key takeaways and intended change reflect the outcome of this study, in the form of plans to seek medical guidance from applicable health services before taking antibiotics. It would be great to have data on AMR knowledge before educating the participants, which would yield a clear conclusion about the knowledge transferred.

Is the work clearly and accurately presented and does it cite the current literature?

Yes

If applicable, is the statistical analysis and its interpretation appropriate?

I cannot comment. A qualified statistician is required.

Are all the source data underlying the results available to ensure full reproducibility?

Yes

Is the study design appropriate and is the work technically sound?

Yes

Are the conclusions drawn adequately supported by the results?

Yes

Are sufficient details of methods and analysis provided to allow replication by others?

Yes

Reviewer Expertise:

Antibiotic discovery

I confirm that I have read this submission and believe that I have an appropriate level of expertise to confirm that it is of an acceptable scientific standard.

# Reviewer report: Reviewer response for version 1

**DOI:** 10.21956/wellcomeopenres.27927.r153003

**Published:** 2026-04-27

**Authors:** Jayshree Dawane

**Affiliations:** 1 Bharati Vidyapeeth (Deemed to be University), Pune, Maharashtra, India

This study presents an innovative and context-sensitive approach to addressing antimicrobial resistance (AMR) through community engagement. The use of a circus-based, co-designed intervention is particularly noteworthy, as it is introduced in culturally familiar and entertaining formats to communicate complex biomedical concepts in an accessible way.

 Minor improvements in methodological transparency and discussion of limitations (e.g., generalizability, potential bias in self-reported behavior change) would further strengthen the manuscript. Acrobatics and Gamification are the best modes to convey the appropriate messages to the community.

Is the work clearly and accurately presented and does it cite the current literature?

Yes

If applicable, is the statistical analysis and its interpretation appropriate?

Yes

Are all the source data underlying the results available to ensure full reproducibility?

Partly

Is the study design appropriate and is the work technically sound?

Yes

Are the conclusions drawn adequately supported by the results?

Yes

Are sufficient details of methods and analysis provided to allow replication by others?

Yes

Reviewer Expertise:

Pharmacology, Herbal medicines, Diabetes

I confirm that I have read this submission and believe that I have an appropriate level of expertise to confirm that it is of an acceptable scientific standard.

# Reviewer report: Reviewer response for version 1

**DOI:** 10.21956/wellcomeopenres.27927.r148506

**Published:** 2026-03-12

**Authors:** Joanna Blackburn

**Affiliations:** 1 University of Huddersfield, Huddersfield, UK

This is a very interesting novel paper and is very well written and presented. There is lots of detailed description of the problem, rationale, methodology and results and the discussion and conclusions raise interesting points for future research. The discussion would benefit from describing the implications for health literacy as presented in the introduction.

Is the work clearly and accurately presented and does it cite the current literature?

Yes

If applicable, is the statistical analysis and its interpretation appropriate?

Yes

Are all the source data underlying the results available to ensure full reproducibility?

Yes

Is the study design appropriate and is the work technically sound?

Yes

Are the conclusions drawn adequately supported by the results?

Yes

Are sufficient details of methods and analysis provided to allow replication by others?

Yes

Reviewer Expertise:

Antimicrobial resistance, antimicrobial stewardship, co-production, shared care, wound care, healthcare professional education, behaviour change.

I confirm that I have read this submission and believe that I have an appropriate level of expertise to confirm that it is of an acceptable scientific standard.
